# Ethical guidelines for publishing in the journal of cachexia, sarcopenia and muscle: update 2017

**DOI:** 10.1002/jcsm.12261

**Published:** 2017-11-03

**Authors:** Stephan von Haehling, John E. Morley, Andrew J.S. Coats, Stefan D. Anker

**Affiliations:** ^1^ Department of Cardiology and Pneumology University of Göttingen Medical Center Göttingen Germany; ^2^ Divisions of Geriatric Medicine and Endocrinology Saint Louis University School of Medicine St Louis USA; ^3^ San Raffaele Pisana Scientific Institute Rome Italy; ^4^ Division of Cardiology and Metabolism – Heart Failure, Cachexia & Sarcopenia; Department of Cardiology (Campus Virchow‐Klinikum); and Berlin‐Brandenburg Center for Regenerative Therapies (BCRT); Deutsches Zentrum für Herz‐Kreislauf‐Forschung (DZHK) Berlin Charité Universitätsmedizin Berlin Berlin Germany

**Keywords:** Ethical guidelines, Publishing

## Abstract

This article details an updated version of the principles of ethical authorship and publishing in the Journal of Cachexia, Sarcopenia and Muscle (JCSM). At the time of submission to JCSM, the corresponding author, on behalf of all co‐authors, needs to certify adherence to these principles. The principles are as follows:

1. All authors listed on a manuscript considered for publication have approved its submission and (if accepted) publication as provided to JCSM.
2. No person who has a right to be recognized as author has been omitted from the list of authors on the submitted manuscript.
3. Each author has made a material and independent contribution to the work submitted for publication.
4. The submitted work is original and is neither under consideration elsewhere nor that it has been published previously in whole or in part other than in abstract form.
5. All authors certify that the work is original and does not contain excessive overlap with prior or contemporaneous publication elsewhere, and where the publication reports on cohorts, trials, or data that have been reported on before these other publications must be referenced.
6. All original research work has been approved by the relevant bodies such as institutional review boards or ethics committees.
7. All conflicts of interest, financial or otherwise, that may affect the authors' ability to present data objectively, and relevant sources of funding have been duly declared in the manuscript.
8. The manuscript in its published form will be maintained on the servers of JCSM as a valid publication only as long as all statements in the guidelines on ethical publishing remain true.
9. If any of the aforementioned statements ceases to be true, the authors have a duty to notify the Editors of JCSM as soon as possible so that the available information regarding the published article can be updated and/or the manuscript can be withdrawn.

The Journal of Cachexia, Sarcopenia and Muscle (JCSM) is a peer‐reviewed international journal dedicated to publishing materials that are related to cachexia and sarcopenia as well as to body composition and its physiological and pathophysiological changes during the lifespan and in different illnesses from all fields of the life sciences. JCSM was founded in 2010 and has attracted interest by both clinicians and researchers, which is buttressed by increasing download numbers, citations, submissions, and by an increasing impact factor.^1^, ^2^ Submissions to JCSM are now coming from and interest in the field of body wasting is embracing the entire globe.^3^, ^4^

The authors of these ethical guidelines, who are editing five peer‐reviewed journals including—besides JCSM—the *Journal of the American Medical Directors Association*, *ESC Heart Failure* and the two new daughter journals *JCSM Clinical Reports* and *JCSM Rapid Communications*, share the view that academic publishing depends, to a great extent, on trust between authors, reviewers, editors, and the academic and lay public.^5^, ^6^, ^7^ Scientific publications are made in an environment of many competing interests that may be commercial (in many different ways), intellectual, and/or political in nature. Some of these competing interests create conflicts of interest. In this context, the Editors of JCSM see it as their duty to promote transparency and research integrity. And, as this is an ongoing effort, we wish to recall the validity and update the statements on ethical publishing made by us before.^8^, ^9^

All authors wishing to publish in JCSM are required to certify that they comply with the guidelines of publishing in JCSM outlined in the succeeding text. This should be performed by stating as part of the published manuscript that ‘All authors of this manuscript comply with the guidelines of ethical authorship and publishing in the Journal of Cachexia, Sarcopenia and Muscle’, providing reference to these guidelines by referencing this article. Specifically, the corresponding author, on behalf of all co‐authors,

1. certifies that all authors listed on the manuscript that is to be considered for publication in JCSM have approved its submission and (if accepted) its publication as provided to JCSM;
2. certifies that no person who has a right to be recognized as author has been omitted from the list of authors on the submitted manuscript;
3. certifies that each author has made a material and independent contribution to the work submitted for publication;
4. certifies that the submitted work is original and is neither under consideration elsewhere nor has been published previously in whole or in part other than in abstract form;
5. certifies that the work is original and does not contain excessive overlap with prior or contemporaneous publication elsewhere; where the publication reports on cohorts, trials, or data that have been reported on before, these other publications must be referenced, and the degree of overlap and difference must be fully described and justified; excessive overlap that is not justified in a cover letter will lead to rejection or withdrawal of the manuscript;
6. certifies that for all original research work, the relevant bodies such as institutional review boards or ethics committees have approved the studies; this rule is not applicable for review articles or editorial comments that give reference to other people's work;
7. certifies that all conflicts of interest, financial or otherwise, that may affect the authors' ability to present data objectively, and relevant sources of funding have been duly declared in the manuscript; and
8. understands that the manuscript in its published form will be maintained on the servers of JCSM as a valid publication only as long as all statements in the guidelines on ethical publishing remain true.
9. If any of the aforementioned statements ceases to be true, the authors have a duty to notify the Editors of JCSM as soon as possible so that the available information regarding the published article can be updated and/or the manuscript can be withdrawn.

We will continue our editorial work for JCSM, and increasing numbers of submissions that have led to increase the numbers of issues of JCSM to now six per year underscore the continuing need for such a niche journal. This is particularly so, because diagnostic tools have improved and overall awareness of cachexia and sarcopenia has increased, however, there still remains a great unmet medical need, because cachexia and sarcopenia are still associated with an unacceptably high burden of morbidity and mortality affecting patients, families, caregivers and healthcare systems.

Despite the existing wish to advance our knowledge, we recognize that the world is not free of scientific misconduct, which can come in many ways. We are happy to report that only very few problems have arisen in the past 7 years, and no published paper had to be withdrawn so far. The Editors of JCSM aim to continue to keep a close eye on these issues, and we need reviewers to help in this. We simply ask that authors play fair to the academic community and the public at large, and then none of this will ever be an issue. We want to take a lead in identifying, notifying, investigating, and reporting all occurrences of scientific misconduct related to JCSM. We will withdraw papers, if significant problems come to our attention. This shall be no surprise to anyone when it has to occur. Hence, we request that these ethical guidelines for publishing in JCSM are cited in each article. As stated before, academic publishing is built on trust—we want JCSM to be a trusted source of best available scientific knowledge in the field. We hope that, together, we can achieve this objective, and our journal can succeed in this ambition.
